# Uncovering the pharmacology of Ginkgo biloba folium in the cell-type-specific targets of Parkinson’s disease

**DOI:** 10.3389/fphar.2022.1007556

**Published:** 2022-09-29

**Authors:** Yu-Chen Yan, Zhi-Heng Xu, Jian Wang, Wen-Bo Yu

**Affiliations:** Department of Neurology and National Research Center for Aging and Medicine and National Center for Neurological Disorders, State Key Laboratory of Medical Neurobiology, Huashan Hospital, Fudan University, Shanghai, China

**Keywords:** Parkinson’s disease, Ginkgo biloba folium, network pharmacology, deep learning, single-nuclei RNA sequencing

## Abstract

Parkinson’s disease (PD) is the second most common neurodegenerative disease with a fast-growing prevalence. Developing disease-modifying therapies for PD remains an enormous challenge. Current drug treatment will lose efficacy and bring about severe side effects as the disease progresses. Extracts from Ginkgo biloba folium (GBE) have been shown neuroprotective in PD models. However, the complex GBE extracts intertwingled with complicated PD targets hinder further drug development. In this study, we have pioneered using single-nuclei RNA sequencing data in network pharmacology analysis. Furthermore, high-throughput screening for potent drug-target interaction (DTI) was conducted with a deep learning algorithm, DeepPurpose. The strongest DTIs between ginkgolides and MAPK14 were further validated by molecular docking. This work should help advance the network pharmacology analysis procedure to tackle the limitation of conventional research. Meanwhile, these results should contribute to a better understanding of the complicated mechanisms of GBE in treating PD and lay the theoretical ground for future drug development in PD.

## Introduction

Parkinson’s disease (PD) is mainly manifested by progressive motor impairment (Armstrong and Okun, 2020), leading to severe damage to the everyday lifestyle of 6.1 million patients worldwide (Shandilya et al., 2022). Prevalence and disability-adjusted life years (DALYs) of PD have been increasing in recent decades (Collaborators, 2019), causing an enormous burden on the medical system and economy (Rocca, 2018).

The development of novel PD therapeutics is in urgent demand. Without available disease-modifying therapy, current treatments for PD are only symptomatic (Vijiaratnam et al., 2021). Long-term symptomatic therapy brings about adverse events, such as dyskinesia and impulse control disorders (Voon et al., 2017).

Herbal medicines, including extracts from Ginkgo biloba extract (GBE), have gradually come to attention as novel therapies for PD. Herbal medicines generally share the advantages of multilevel functions with fewer adverse effects (Yin et al., 2021). Among the most frequently applied herbal medicines, GBE has been used in clinical therapies since the early 1970s (Saponaro et al., 1971; Bartolo, 1973). One of the most investigated applications of GBE is in treating neurodegenerations, represented by Alzheimer’s disease and mild cognitive impairment (Singh et al., 2019; Nowak et al., 2021; Tomino et al., 2021). Various studies have also validated that a mixture of GBE exerts neuroprotective function on both *in vivo* and *in vitro* PD models, including toxin-induced PD models on rats (Yu et al., 2021), toxin-induced PD mice (Rojas et al., 2009; Rojas et al., 2012), transgenic PD mice (Kuang et al., 2018), and *in vitro* cultured cell models (Yang et al., 2001; Kang et al., 2007). Subsequent research is hindered by the mixture nature of GBE and its multi-target effects. A complicated extraction procedure is required to obtain bioactive components from GBE (Liu et al., 2022; Ma et al., 2022), and the procedure is still ongoing improvement (Boateng, 2022). The variability of extracts results in difficulty in repeating results across studies, thereby hampering the exploration of molecular mechanisms. Delineating the effects of a single active component in GBE would contribute to proposing feasible targets and aiding future drug development for PD.

Herein, advances in analytical pharmacy and bioinformatics would help to detangle the complex molecular mechanisms underlying the therapeutic efficacy of GBE in PD. The single-nuclei RNA sequencing (snRNA-seq) has emerged as a powerful tool for identifying and characterizing cell types, states, and lineages (Slyper et al., 2020). Recently, the snRNA-seq approach was conducted to analyze the transcriptome in midbrains of PD patients (Smajić et al., 2022). Therefore, we took the unprecedented chance to investigate GBE effects in a cell-type-specific manner. We intended to focus on microglia and astrocytes in addition to neurons when studying the effects of GBE. Since PD is attributed to a selective loss of dopaminergic neurons in the substantia nigra. Meanwhile, mounting clinical and experimental evidence illuminated that glial cells, especially microglia and astrocytes, were not only responders but also significant mediators in PD pathogenesis (Sorrentino et al., 2019; Bartels et al., 2020). Thus, modulating microglia and astrocytes functions is a promising pharmacological strategy for treating PD (Grotemeyer et al., 2022; Lee et al., 2022).

The deep learning approach can be another handy tool to guide pharmacological studies, including drug-target prediction, drug repurposing, and novel drug discovery (Zhavoronkov et al., 2019; Issa et al., 2021; Zhu et al., 2021). Experimental measurement of the compound–protein binding affinity remains the most accurate method for studying drug-target interactions. However, conventional methods are costly, time-consuming, and laborious, which are infeasible for investigating the multifarious drug-target interactions (DTI) between complex GBE ingredients and numerous PD targets. Therefore, deep learning has been used to conduct high throughput DTI analyses, which could help to screen out potent DTI between GBE ingredients and PD-related bio-targets.

In this study, we tended to identify active components in GBE for PD along with its cell-type-specific targets. Network pharmacology analysis was conducted, integrating data from snRNA-seq and existing drug datasets. A cell-type-specific compound-target-pathway network was established, and DTI was subsequently investigated with a deep learning algorithm. Then, we validated the results by molecular docking. This research will contribute to a better understanding of the molecular mechanisms of treating PD with GBE.

## Methods

### Collecting and selecting compounds in GBE

Firstly, components of GBE were collected via searching the terms: “ginkgo folium,” “folium ginkgo,” and “Yinxingye” in databases. TCMSP (Ru et al., 2014) (https://old.tcmsp-e.com/index.php, version 2.3), TCMID (Huang et al., 2018) (http://bidd.group/TCMID/, version 2.0) and SymMap (Wu et al., 2019) (http://www.symmap.org/, version 2.0) databases rendered 307, 94 and 319 ingredients of GBE, respectively. All data were collected on 18 May 2022.

Secondly, PubChem CID was retrieved from PubChem (https://pubchem.ncbi.nlm.nih.gov/) to identify each component.

We also searched the PubMed database with the following terms " (Ginkgo biloba leaf OR Ginkgo biloba folium) AND (components OR ingredients OR metabolite)" and added ginkgolide K, which was not timely updated in databases (Li et al., 2018a).

Thirdly, chemical properties and pharmacokinetic profiles of components were retrieved. The chemical properties of components were annotated via SwissADME (http://www.swissadme.ch) (Daina et al., 2017), which provided information on molecular weight, lipophilicity (log *P*_o/w_), number of H-bond acceptors, number of H-bond acceptors, number of rotatable bonds, and topological polar surface area (TPSA). ADMETlab 2.0 (Xiong et al., 2021) (https://admetmesh.scbdd.com/) was employed to evaluate compound pharmacokinetics and toxicity. To assess components’ oral bioactivity, HobPre (www.icdrug.com/ICDrug/ADMET) (Wei et al., 2022), a classification model, was exploited. The ADMET profiles of components were obtained from pkCSM (Pires et al., 2015) (http://structure.bioc.cam.ac.uk/pkcsm).

With all the above data collected, Lipinski’s Rule (Lipinski et al., 2001) was subjected to assess the draggability of collected compounds. A total of 25 selected compounds were selected and listed in Supplementary Table S1. These compounds met the following criteria: molecular weight of fewer than 500 Da; log *P*_o/w_ lower than five and higher than −2; five or fewer hydrogen bond donor sites and tenor fewer hydrogen bond acceptor sites; the number of rotatable bonds less than 10.

### Acquiring potential molecular targets of GBE components

Every selected component has been searched in SymMap (Wu et al., 2019) (http://www.symmap.org/, version 2.0) database, and 272 potential molecular targets were retrieved. SymMap database integrates target information from HIT (Ye et al., 2011) (http://lifecenter.sgst.cn/hit/), TCMSP, HPO (Köhler et al., 2021) (https://hpo.jax.org/app/), DrugBank (Wishart et al., 2018) (https://go.drugbank.com/), NCBI(https://www.ncbi.nlm.nih.gov/) and HERB (Fang et al., 2020a) (http://herb.ac.cn/) databases. Additional pharmacoproteomic and pharmaco-transcriptomic data were obtained manually. Additional ginkgolide J, ginkgolide M, and ginkgolide K targets data, which is not included in the above databases, was retrieved from the Comparative Toxicogenomics Database (CTD) (Davis et al., 2017) (URL: http://ctdbase.org/). All data were collected on 18 May 2022. After removing duplicates, 283 genes were identified as putative GBE targets for PD.

### Acquiring PD-related-targets in different cell types from single-nuclei RNA sequencing data

Gene expression profile of different cell types from the idiopathic Parkinson’s disease patient’s brain snRNA-seq (Smajić et al., 2022) (GSE157783) was used to identify the disease-related targets in this study. Cell-type-specific genes were identified using the Quasi-Poisson generalized linear model implemented in the fit models function of the R package monocle3 (version 1.0.0) (Trapnell et al., 2014). The cutoff q coefficient was set at 0.05 to obtain differentially expressed genes (DEGs) in each cell type. The potential targets were identified by overlapping genes of GBE targets and DEGs in different cell types of PD. Intersections were visualized with R package VennDiagram (version 1.7.3) (Chen and Boutros, 2011).

### PPI networks construction

Protein-protein interaction (PPI) network of all targets was constructed using Cytoscape software (version 3.9.1) with data from STRING (Szklarczyk et al., 2015) (version 10.0) database. The confidence score cutoff was set at 0.4.

### GO and KEGG pathway enrichment analysis

R package topGO (version 2.46.0) and cluster profile (version 4.2.2) was employed to conduct Gene Ontology (GO) and KEGG pathway analysis. Reference gene data were retrieved using R package, org. Hs.eg.db (version 3.14.0). The *p*-value cutoff was set at 0.05, and the q-value cutoff was set at 0.01 for all analyses. Top clusters from GO and KEGG enrichment were visualized using R package ggplot2 (version 3.3.5) and enrichplot (version 1.14.2). All mentioned analysis was conducted on R version 4.1.2.

### Drug-target interaction (DTI) prediction with DeepPurpose

Pre-trained model CNN_CNN_BindingDB provided by DeepPurpose (Huang et al., 2020) (https://github.com/kexinhuang12345/DeepPurpose) was used to calculate the binding score between selected targets and their proven ligands. In this pre-trained model, Convolutional Neural Network (CNN) was chosen to encode SMILES of components and the amino acid sequence. The Binding Database (BindingDB), a public drug-target binding benchmark dataset, was employed to provide measured binding affinities. DeepPurpose generates predictions via a Multi-Layer Perceptron (MLP), one of the most common artificial neural networks. All amino acid sequences of the selected targets were collected from UniProt (Consortium, 2020) (https://www.uniprot.org/). The SMILES of each component were obtained from the PubChem database (https://pubchem.ncbi.nlm.nih.gov/).

### Molecular docking

Molecular docking was performed using the SwissDock (Grosdidier et al., 2011) server (http://www.swissdock.ch/). 3D structure of MAPK14 protein was obtained from RCSB PDB (https://www.rcsb.org/) with PDB ID: 1WBS. The chemical structure of ginkgolide J and ginkgolide A was obtained from the PubChem database (https://pubchem.ncbi.nlm.nih.gov/). The DockPrep plugin of Chimera (version 1.16, build 42,360) was employed to prepare the structures before docking. Docking results were analyzed and visualized using UCSF Chimera (version 1.16, build 42,360) and LigPlot (Laskowski and Swindells, 2011) (version 2.2.5).

## Results

### Potential active components and related targets of GBE

**FIGURE 1:**
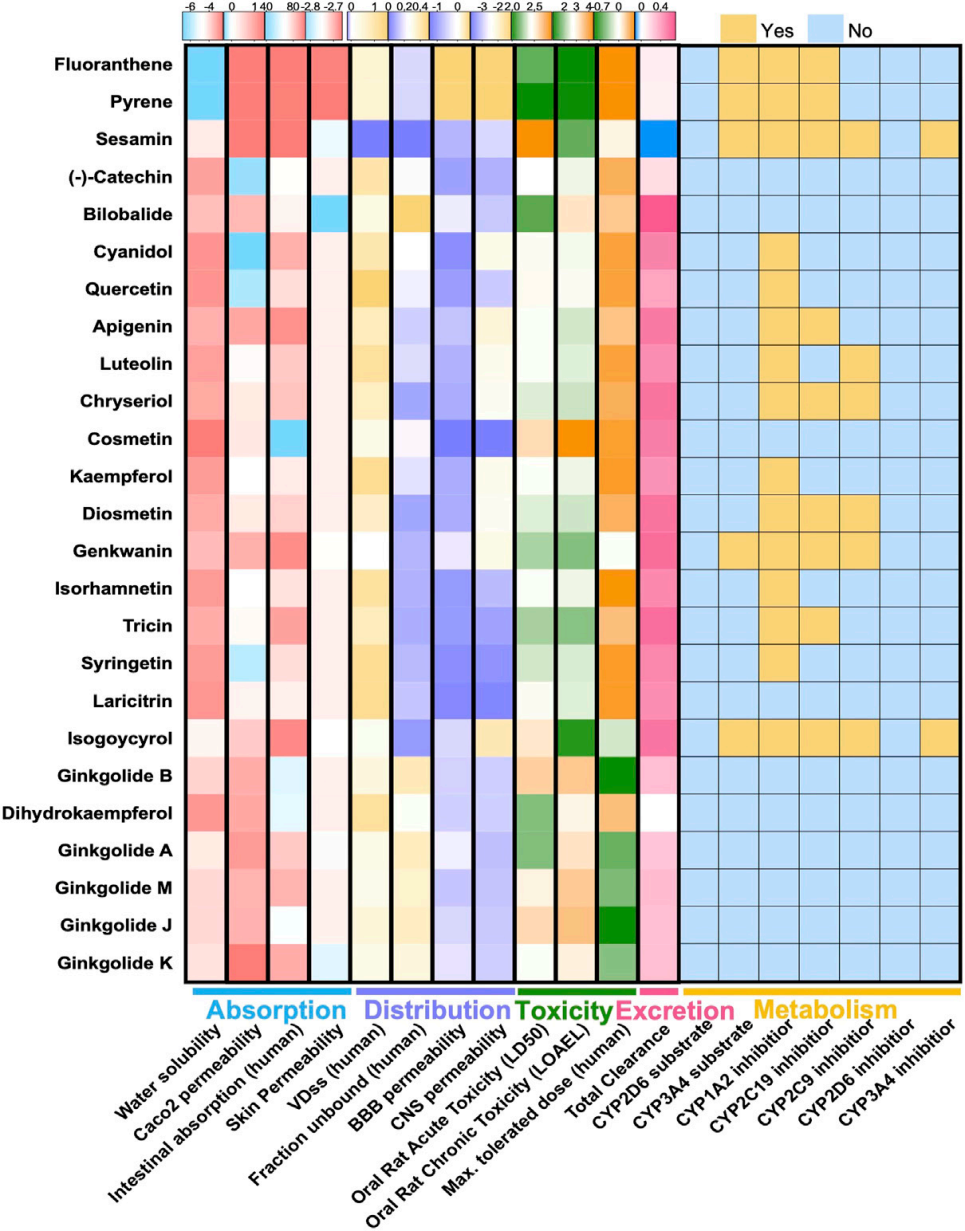
ADMET features of 26 compounds in GBE. Heatmaps showed pharmacokinetic parameters of two components in GBE, including parameters describing drug absorption, distribution, metabolism, excretion, and toxicity.

Chemical components of GBE were searched and collected from TCMSP, TCMID, and SymMap databases and manually checked references from PubMed. After screening the druggability of these compounds using Lipinski’s Rule (Lipinski et al., 2001), a total of 25 compounds were selected (Figure 1). The selected components’ chemical properties and ADMET profiles were listed in detail (Supplementary Table S1). Flavonoids or flavonoid derivatives were the major part of GBE. Other major active compounds were the terpenoid of the Ginkgo biloba, including bilobalide and ginkgolides. Most components were with high permeability, indicating a high degree of absorption. The toxicity of all components was relatively low except for fluoranthene and pyrene, which suggested suitability for drug development. Targets of these selected compounds were retrieved from databases and supplemented by manually screened references, rendering 283 potential targets.

### Target genes in different cell types of PD

**FIGURE 2:**
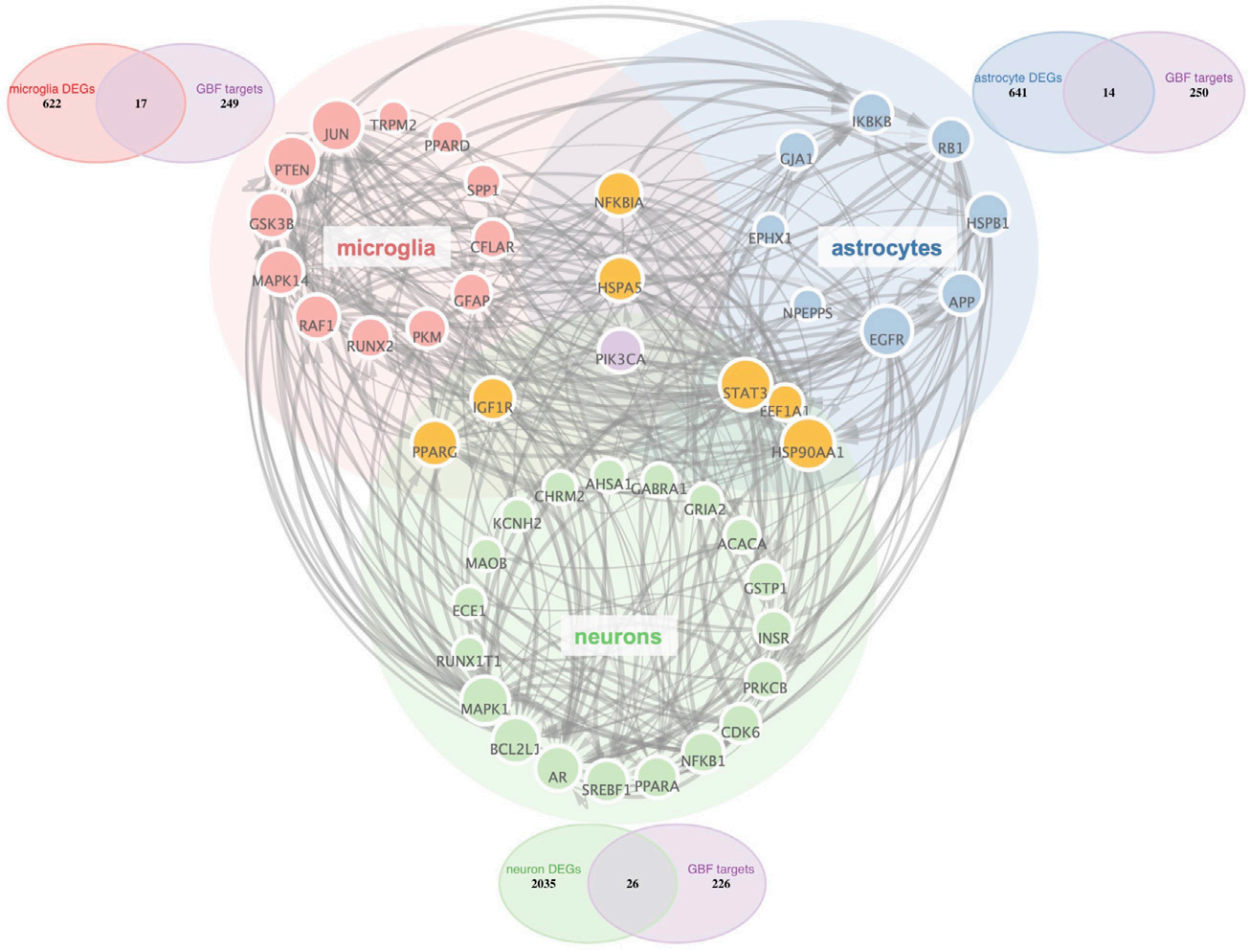
PPI Network analysis of potential GBE targets for PD. All GBE targets for PD in microglia, neurons, and astrocytes were identified via PPI Network. Node size was positively correlated to the degree score. The thickness of the edges indicated the connectivity score between linking nodes. Three Venn diagrams showed cell-type-specific targets in the intersection between PD-related targets and GBE targets.

Aided by recent advances in the snRNA-seq technique, we could characterize all cell types in the midbrain of Parkinson’s disease (Smajić et al., 2022). Astrocytes and microglia have been proved critical modulators in PD pathogenesis and are both targets of disease-modifying therapies (Bartels et al., 2020; Lee et al., 2022). Thus, we selected microglia, astrocytes, and neurons as potential cellular targets of GBE. PD-related targets were determined according to DEGs in different cell types. As shown in Venn diagrams (Figure 2), potential targets were identified in the intersections between GBE and PD-related targets. The 3-category Veen diagram showed that only several targets were shared between cell types, such that PIK3CA was identified as a potential target in all 3 cell types. Despite that, most GBE targets were unique in each cell type. Cell-type-specific GBE targets were listed in Supplementary Table S2, and Supplementary Figure S1 showed the overlapping status of GBE targets in other cell types.

### GO and KEGG pathway enrichment analysis

**FIGURE 3:**
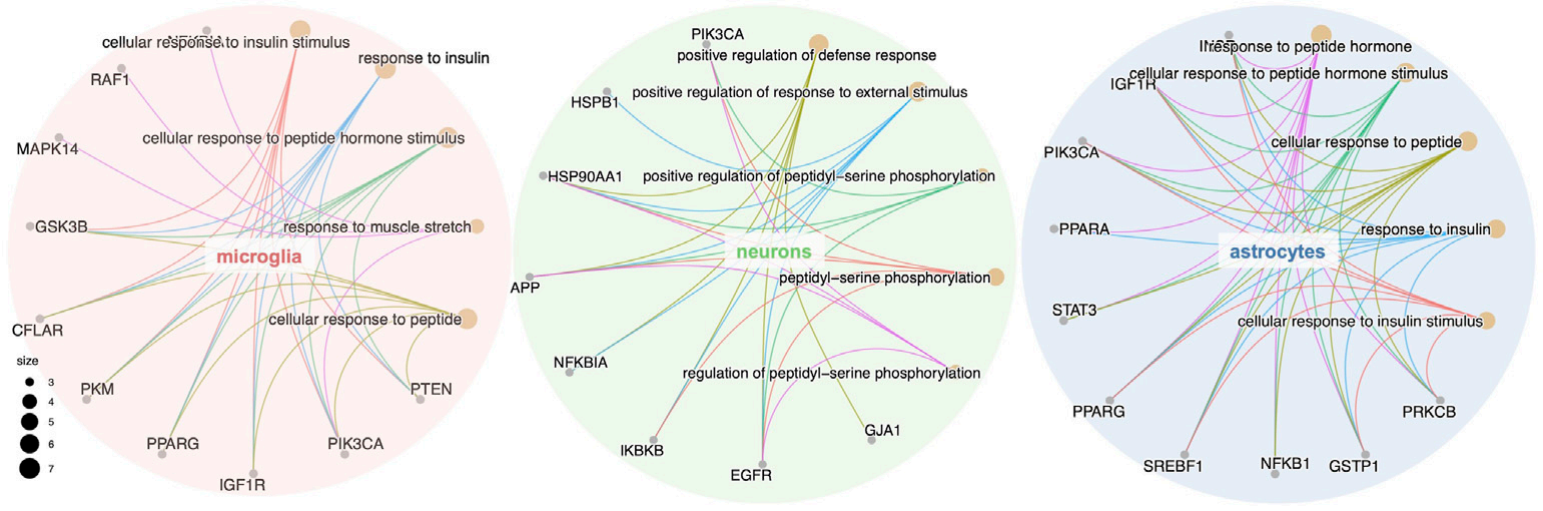
GO enrichment analysis of the potential GBE targets for PD in three cell types. GO enrichment analysis of the potential GBE targets in microglia, neurons and astrocytes were conducted separately. The top five categories of biological process (BP) for each cell type were shown together with genes enriched in each category. Dot size represents the number of genes enriched in each category.

**FIGURE 4:**
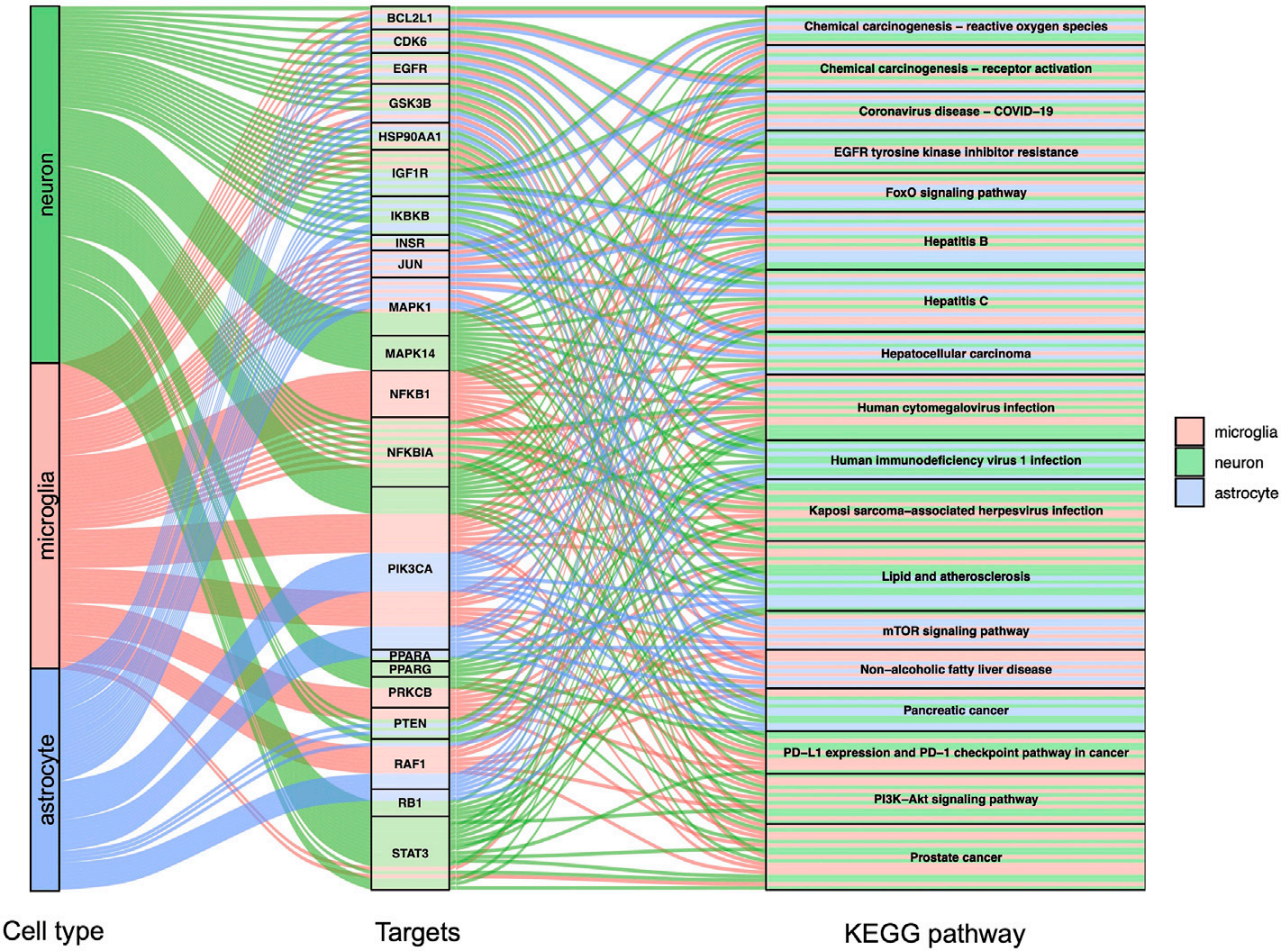
KEGG enrichment analysis for potential GBE targets for PD. Sankey diagram showed top enriched KEGG pathways and according to targets in 3 cell types. *p* < 0.0015 in all shown pathways.

**FIGURE 5:**
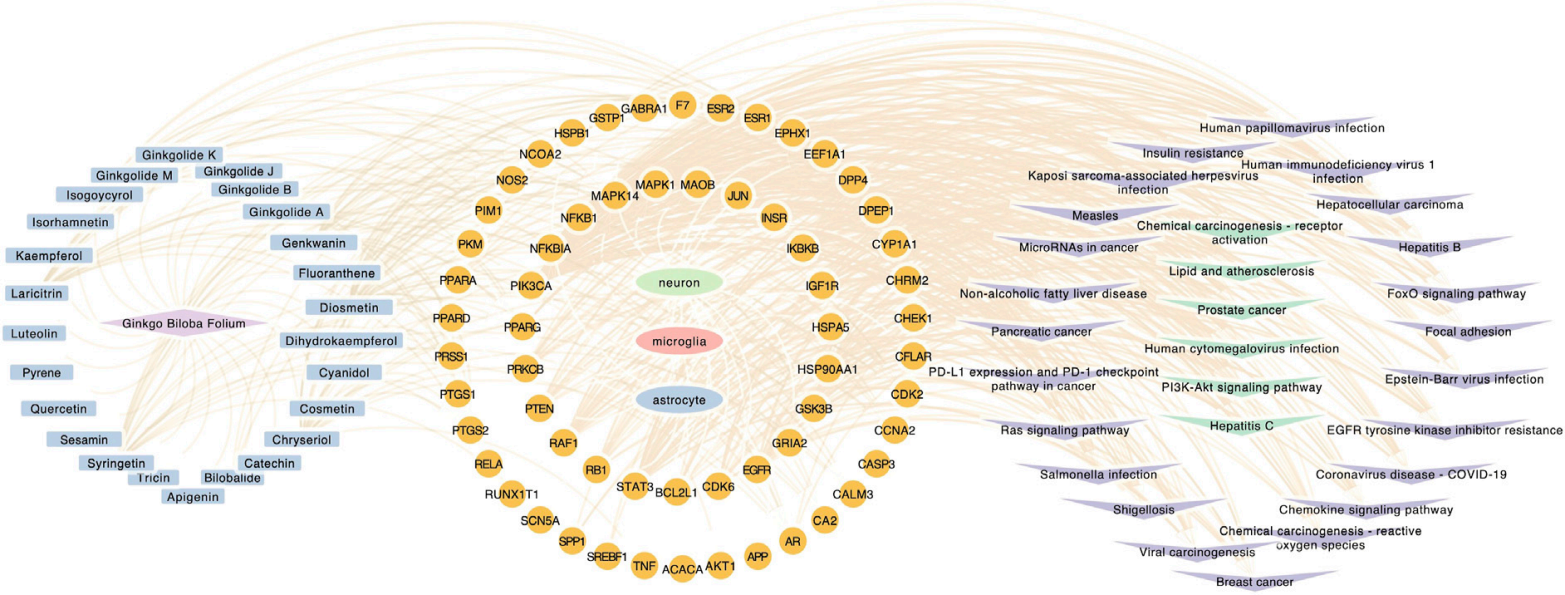
Component-target-pathway network. Component-target-pathway were holistic integrated into the network diagram. Blue nodes in the left circle represent potential active components in GBE. Yellow nodes in the middle circle represent PD targets linking to specific cell types. Purple and green nodes on the right circle represent correlating pathways.

GO, and KEGG enrichment analysis was performed separately on GBE targets in 3 cell types, documented in Supplementary Tables S3, S4, respectively. The categories of pathway enrichment include biological process (BP), cellular component (CC), and molecular function (MF). The top enriched BP of targets is shown in Figure 3 and Supplementary Figure S2. Note that no CC category was enriched with a *p*-value set at 0.05 in microglia targets. Results showed that GBE potentially contributes to PD therapy by influencing neurons on biological processes involved in the cellular response to peptide hormone and peptide. For astrocytes, GBE would affect biological processes relating to peptidyl-serine phosphorylation. In microglia, GBE may also contribute to peptidyl-serine modification and response to insulin. Figure 4 and Supplementary Figure S3 illustrated the top KEGG pathways with most genes enriched. Top enriched pathways concerned targets from more than one single cell type. Most high-rank pathways were related to viruses, including coronavirus, hepatitis, measles, human cytomegalovirus, and human immunodeficiency virus 1. Tumor-associated pathways were also enriched concerning hepatocellular carcinoma, pancreatic cancer, prostate cancer, and PD-1 checkpoint pathway in cancer. Since PD-related pathways like the mTOR signaling pathway and PI3K-Akt signaling pathway were also enriched, these analysis results indicated an overall effect concerning multiple signal pathways of GBE to treat PD. GBE may influence several vital pathways in PD by influencing more than one cell type. Figure 5 shows a holistic integration of drug-components-target-pathway interactions.

### DTI prediction with DeepPurpose and molecular docking

**FIGURE 6:**
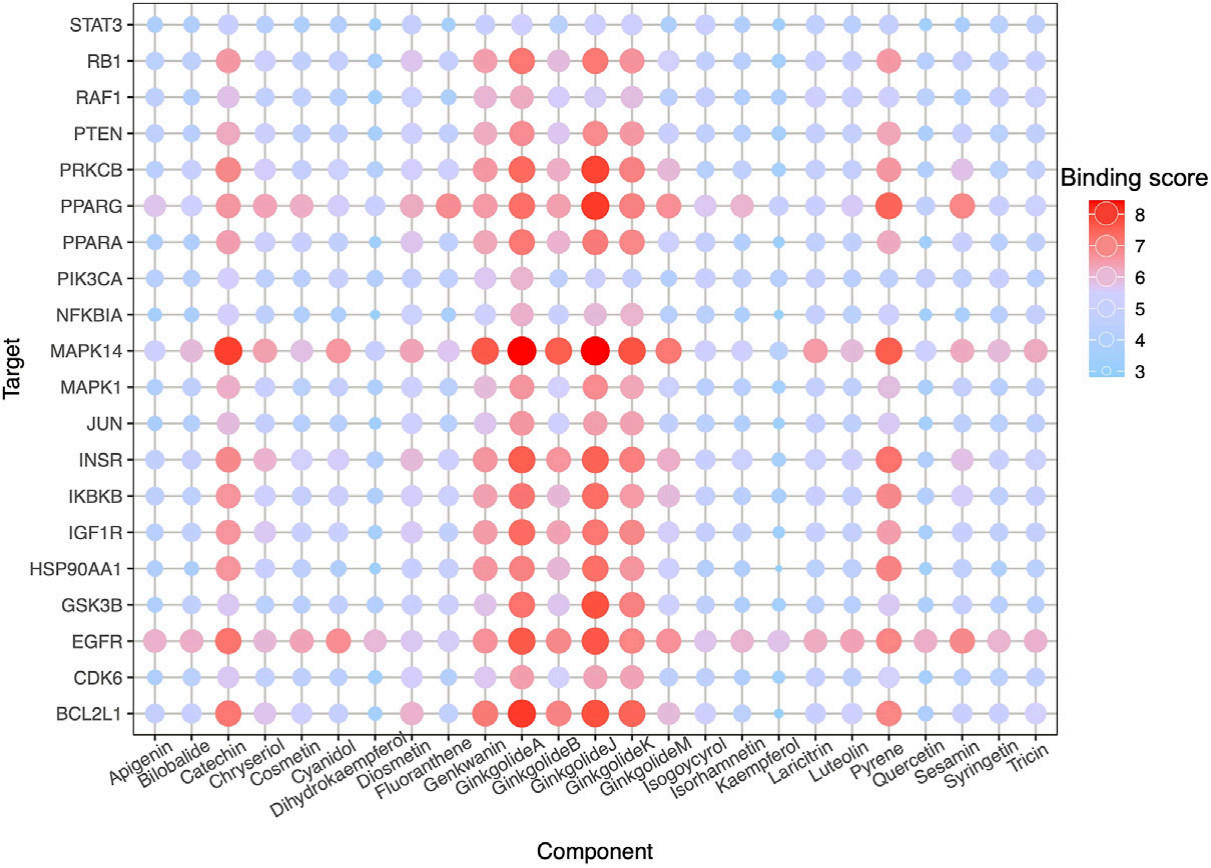
DTI prediction. Dot-matrix showed a binding score predicted using the cnn_cnn_bindingdb model, a pre-trained deep mode of DeepPurpose. The color and size of dots indicate the level of binding score.

**FIGURE 7:**
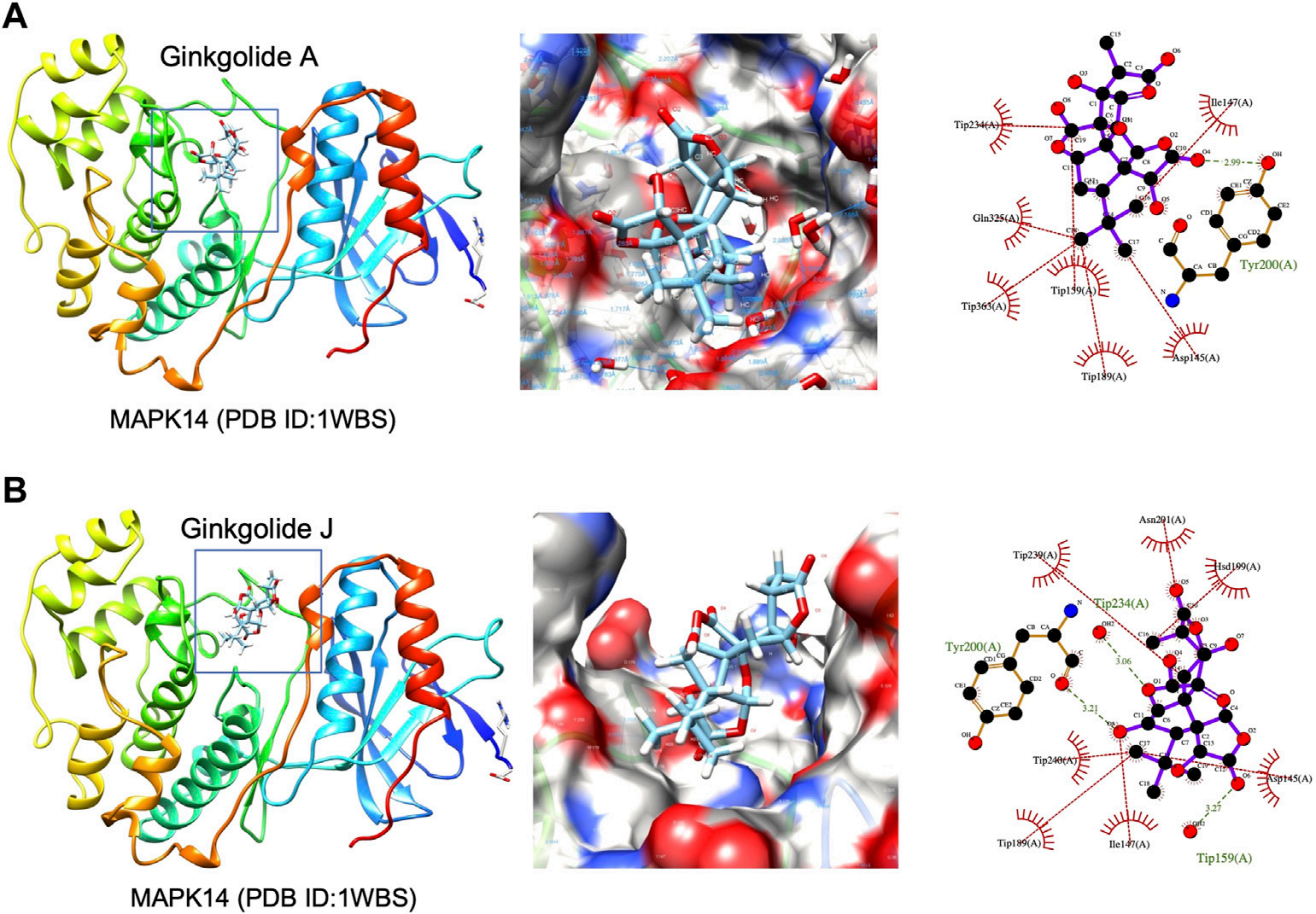
3D structures of MAPK14 docked with ginkgolide A or ginkgolide J. **(A)** Docking of ginkgolide A to MAPK14(PDB ID:1WBS). Left: 3D structure represented of ginkgolide A docked to MAPK14. Middle: Surface representation of ginkgolide A docked to MAPK14. Right: Protein-ligand interaction visualized by Ligplot. **(B)** Docking of ginkgolide J to MAPK14(PDB ID:1WBS). Left: 3D structure represented of ginkgolide J docked to MAPK14. Right: Surface representation of ginkgolide J docked to MAPK14. Right: Protein-ligand interaction visualized by Ligplot.

High-throughput analysis was conducted to predict the potential drug‐target interaction (DTI) between all 25 active compounds in GBE and all 47 PD targets in three cell types. Detailed results are listed in Supplementary Table S5. Predicted binding scores of targets in top enriched pathways were shown in the matrix plot in Figure 6. The binding score between MAPK14 and ginkgolide J was 8.43, the highest among all predicted, suggesting a possible strong interaction between them. The second highest interaction between MAPK14 and ginkgolide A reached 8.41. Notably, ginkgolides A, B, J, and K showed similar binding patterns to potential targets, possibly due to similar chemical structures among these compounds. Consequently, molecular docking was performed to confirm the affinity between MAPK14 and ginkgolide J or A. As shown in the 3D and 2D structures, MAPK14 will be stably docked with ginkgolide J or ginkgolide A, and the delta G calculated for each docking were −7.207,632 kJ/mol and −7.0555,134 kJ/mol, respectively (Figure 7). Molecular docking results were in agreement with predictions rendered by DeepPurpose. Detailed results of molecular docking are listed in Table 6. In conclusion, DTI between selected targets and GBE components was predicted with DeepPurpose. Further validation with the molecular docking approach suggested a potentially strong interaction between ginkgolide J or A with MAPK14, a potential target for PD.

## Discussion

Parkinson’s disease (PD) is a neurodegenerative disorder due to selective loss of dopaminergic neurons in the substantia nigra and Lewy body formation (Kalia and Lang, 2015). PD patients suffer from motor-dominant symptoms, including tremors at rest, bradykinesia, stiffness, and postural instability (Movement Disorder Society Task Force on Rating Scales for Parkinson’s Disease, 2003). Non-motor signs severely diminish the life quality of PD patients as well. For example, hyposmia, rapid eye movement (REM), sleep behavior disorder (RBD), depression, and constipation can precede the symptoms related to dopamine deficiency for several years or arise later in the disease (Schapira et al., 2017).

Developing effective therapeutics to slow or halt the progression of PD remains a top priority for researchers. So far, no agents have been proven with sufficient evidence for disease-modifying effects in PD (Lang and Espay, 2018; Vijiaratnam et al., 2021). Currently used therapies alleviate symptoms initially while losing efficacy as the disease progresses (Beckers et al., 2022). Furthermore, dopaminergic medications bring about motor and non-motor behavioral side-effects (Voon et al., 2017). Approximately 80% of PD patients on levodopa treatment would suffer from drug-induced dyskinesia (Espay et al., 2018; Olanow et al., 2020). Long-term application of medication also results in impulse control disorders, including gambling disorder, binge eating disorder, compulsive sexual behavior, and compulsive shopping (Voon et al., 2017).

Natural herbal medicines like extracts from Ginkgo biloba extract (GBE) have shed light on drug development for PD. Herbal products have gradually gained acceptance in treating neurodegenerative diseases for their multi-functional characteristic with relatively fewer adverse effects (Wahid et al., 2020; Gregory et al., 2021; Wang et al., 2021). Herbal medicines have been applied to treat PD (Chen et al., 2022; Sharma et al., 2022; Zahedipour et al., 2022), and GBE has proven efficacy. *G. biloba*, a medicinal plant belonging to the Ginkgoaceae family, is considered the oldest tree alive in the world (Chen et al., 2021). GBE has been used for medical purposes for centuries in various diseases, typically for cardiovascular conditions (Li et al., 2018b; Zhan et al., 2021; Tao et al., 2022). Commercialized GBE, EGB 761^®^, has a recognized neuroprotective role for cognitive impairment like Alzheimer’s disease (AD) (Liu et al., 2019; Verma et al., 2020; Abdelmeguid et al., 2021; Ge et al., 2021; Zhao et al., 2021). In many European states, EGB 761®is the only drug therapy in the guideline for treating mild cognitive impairment (MCI) (Kandiah et al., 2019; Tomino et al., 2021; Barbalho et al., 2022). Various studies have provided experimental evidence supporting GBE, a mixture of active components, as a competent intervention for alleviating PD (Kuang et al., 2018; Mohammed et al., 2020; Yu et al., 2021). Existing evidence supported that GBE alleviates neuroinflammation (Mohammed et al., 2020) and oxidative impairments (Kuang et al., 2018; Mohammed et al., 2020) in PD models. The Akt/GSK3β pathway may be involved in the neuroprotective effects of GBE (Yu et al., 2021).

However, GBE composition may vary across studies, largely dependent on the complicated extraction procedure (Fang et al., 2020b; Boateng, 2022; Liu et al., 2022; Ma et al., 2022).

For a further in-depth understanding of molecular mechanisms, studies attempt to delineate the role of a single active component in GBE. Previous studies have separately focused on several GBE components and validated their protective effects on PD models. These components, including ginkgetin (Wang et al., 2015), amentoflavone (Cao et al., 2017), Ginkgolide B (Liu et al., 2020; Zhao et al., 2020), Ginkgolide K (Yu et al., 2018; Miao et al., 2022), protocatechuic acid (Zhang et al., 2015; Gallardo-Fernández et al., 2019), apigenin (Liu et al., 2015; Anusha et al., 2017), and bilobalide (Hua et al., 2017), have all been proved neuroprotective in PD models when applied alone. These studies revealed that GBE exerted neuroprotective function via various cellular and molecular pathways. The inflammation-related mechanism was among the most investigated (Spagnuolo et al., 2018). p-NF-kB/p65 was regulated by several components in GBE, such as ginkgolide K^83^, protocatechuic acid, and chrysin (Zhang et al., 2015). Another broadly accepted mechanism was relieving oxidative stress (Siima et al., 2020; Behl et al., 2022). Other identified mechanisms included promoting neurotrophic factors like BDNF (Song et al., 2022) and increasing the expression of anti-senescences proteins like SIRT-2 (Gallardo-Fernández et al., 2019).

In this study, bioinformatics approaches were exploited to tackle the limitation of conventional research. Laboratory experiments are hindered by high costs, consuming enormous time and laborious work. Determined by the complex mixture nature of GBE and multifarious targets of PD, laboratory experiments are infeasible to uncover all possible molecular mechanisms underlying drug efficacy. Herein, network pharmacology analysis would guide further investigations by screening potential targets related to bioactive GBE components. The single-nuclei RNA sequencing (snRNA-seq) technique was recently developed and applied to study the midbrain transcriptome of PD patients. This cell-specific data allowed the chance to delineate GBE effects in different cell types. Moreover, advances in deep learning algorithms conferred the potential to conduct high-throughput screening for potent drug-target interaction (DTI) between GBE components and related PD targets.

A total of 25 potentially active components were obtained after selection by Lipinski’s Rule of Five. As mentioned above, part of the ingredients has already been reported for efficacy in PD. Components information was manually curated and supplemented from published reports. Certain ingredients like ginkgolide K were not documented in existing compounds databases since they were not identified as components of GBE until recently (Yuan et al., 2008). With advances in chemistry technologies, ingredients with only trivial amounts in GBE would be detected more thoroughly. Consequent analysis of the pharmacological characteristics would be needed to conduct biological studies.

Mainly, selected GBE compounds largely constitute flavonoids and flavone derivates, such as luteolin, kaempferol, and apigenin. As the main ingredients of GBE, flavonoids are among the most studied herbal products in medical applications (Dong et al., 2022; Tian et al., 2022). Also, our results were consistent with conclusions drawn from studies on PD animal models, which proved flavonoids’ efficacy for neuroprotection (Siima et al., 2020; Rahul and Siddique, 2021). Consequently, rising interest has extensively engrossed in the clinical trial designs of applying flavonoids (Zhang et al., 2022) based on its commonly recognized functions concerning anti-oxidation (Behl et al., 2022) and anti-inflammation (Spagnuolo et al., 2018). Results from this study further support the application of a single component or mixture of flavonoids to PD.

In order to identify GBE targets in a cell-type-specific manner, we analyzed snRNA-seq data on PD midbrain samples (Smajić et al., 2022). Since previous studies validated astrocytes and microglia as significant modulators in PD pathogenesis (Bartels et al., 2020; Lee et al., 2022), subsequent analysis was conducted on astrocytes and microglia in addition to neurons. Most targets of GBE were unique in each cell type. Except that only a few targets were found in intersections between different cell types, such as PIK3CA. At the same time, GO enrichment analysis rendered similar results in astrocytes and microglia. Data suggested that GBE may exert neuroprotection via modulating cellular response to peptide hormone, peptide, and insulin, as well as biological processes relating to peptidyl-serine phosphorylation in glia. KEGG enrichment analysis showed commonly shared pathways across cell types. In all three cell types, pathways related to virus and tumor were significantly enriched, concerning hepatocellular carcinoma, pancreatic cancer, prostate cancer, and PD-1 checkpoint pathway in cancer. An apparent virus and tumor-associated bias in enrichment analysis were observed. One possible explanation was that the GBE targets collected in this study were from existing reports, which have intensively studied its anti-infection and anti-tumor effects (Man et al., 2012; Jiao et al., 2016; Ibrahim et al., 2021). Apart from that, GBE’s anti-oxidative and anti-inflammatory effects have been pivotal to attention in previous studies (Rogerio et al., 2010; Jiao et al., 2016; Lichota et al., 2019). Both play vital roles in infection and tumor-related biological processes (Fridman et al., 2022; Zuo et al., 2022), and both have been recognized for participating in PD pathogenesis (Pajares et al., 2020; Kip and Parr-Brownlie, 2022; Shandilya et al., 2022). Other closely PD-related pathways like the mTOR (Ceccariglia et al., 2020) signaling pathway and PI3K-Akt (Jin et al., 2022; Neves et al., 2022) signaling pathway were also enriched. Collectively, GBE may exert an overall effect concerning different cell types and multiple signal pathways to treat PD.

The component-target-pathway network showed that potential mechanisms were complex interactions between multiple components, targets, and pathways in GBE therapy for PD. Nearly all potentially active components were linked with more than one target for PD. Similarly, most potential targets were regulated by multiple components in GBE.

Herein, we exploited recently developed deep learning technology to help detangle complex drug-target interactions. DTI between all potential compounds and targets was predicted with DeepPurpose, a deep learning method-based approach for drug discovery. Our results suggested relatively strong interactions between ginkgolides and several PD targets participating in core biological pathways. Notably, ginkgolide A, B, J, and K showed similar binding patterns to targets, possibly due to their similar chemical structures.

Published experimental reports supported the reliability of our bioinformatic methods. For instance, a recent study on lipopolysaccharide (LPS) induced inflammation models has shown ginkgolide A as a modulator for MAPK (Li et al., 2017). According to our results from DeepPurpose, predicted binding scores between MAPK14 and ginkgolide A were the second-highest among all tested. Interestingly, our data indicated a more vital interaction between MAPK14 and ginkgolide J than ginkgolide A. Since a higher binding score between ginkgolide J and MAPK14 was predicted by DeepPurpose, corroborating with results by molecular docking approach. Although interactions between ginkgolide J and MAPK had not been reported in experimental reports yet by the time we conducted this bioinformatic study. Supported by published reports and our bioinformatics data, we cautiously proposed that the GBE component, ginkgolide J, may interact with MAPK14 and exert biological function. Conclusions from this study do come with many caveats due to a lack of validation by benchwork experiments. *In vivo* or/and *in vitro* laboratory work is still required to establish concrete interaction between predicted DTI in this study.

## Conclusion

Taken together, through the integration of data from snRNA-seq and employing a deep learning algorithm, a cell-type-specific targets and compound network was established. This work took advantage of recently advanced bioinformatics approaches. Herein, an unprecedented procedure of conducting network pharmacology analyses in a cell-type-specific manner was established. This work will better facilitate our understanding of GBE mechanisms in treating PD. Moreover, identified interaction of drugs and targets would lay a theoretical foundation for the development PD drugs.

## Data Availability

The datasets presented in this study can be found in online repositories. The names of the repository/repositories and accession number(s) can be found in the article/Supplementary Material.
