# Corrigendum: Uncovering the pharmacology of Ginkgo biloba folium in the cell-type-specific targets of Parkinson’s disease with a deep learning algorithm

**DOI:** 10.3389/fphar.2022.1115043

**Published:** 2023-01-12

**Authors:** Yu-Chen Yan, Zhi-Heng Xu, Jian Wang, Wen-Bo Yu

**Affiliations:** Department of Neurology and National Research Center for Aging and Medicine & National Center for Neurological Disorders, State Key Laboratory of Medical Neurobiology, Huashan Hospital, Shanghai, China

**Keywords:** Parkinson’s disease, Ginkgo biloba folium, Network pharmacolgy, deep learning, Single-nuclei RNA sequencing

In the published article, there was an error in the Funding statement. The Funding statement was not present in the original version. The correct Funding statement appears below.

“This work was financially supported by the National Natural Science Foundation of China (Award number(s): 81971194, 82171421, 91949118) and Science and Technology Commission of Shanghai Municipality (Award number(s): 2018SHZDZX01, 21S31902200); National Health Commission of the People’s Republic of China.”

The authors apologize for this error and state that this does not change the scientific conclusions of the article in any way. The original article has been updated.

