# Supplementary figures and images for: Uncovering the pharmacology of Ginkgo biloba folium in the cell-type-specific targets of Parkinson’s disease
Source: Front Pharmacol. 2022 Sep 29;13:1007556. doi: 10.3389/fphar.2022.1007556 (PMC9556873; doi:10.3389/fphar.2022.1007556)

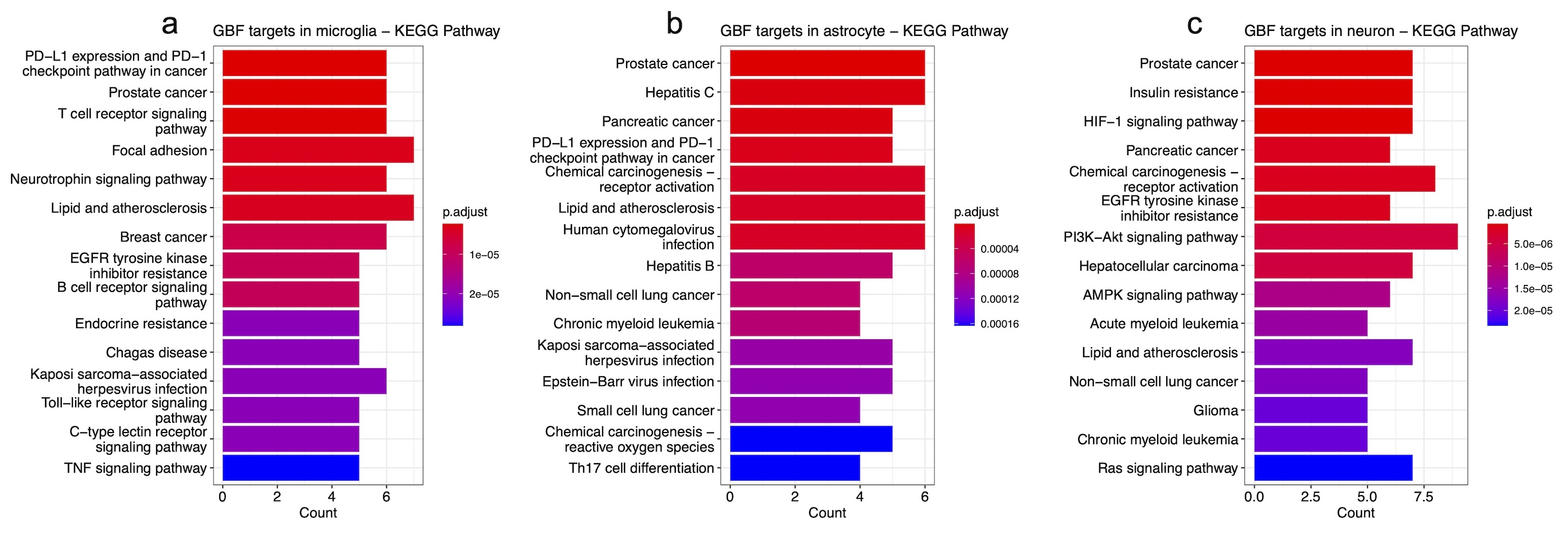

Supplement: Supplementary file 2 [file Image3.JPEG]

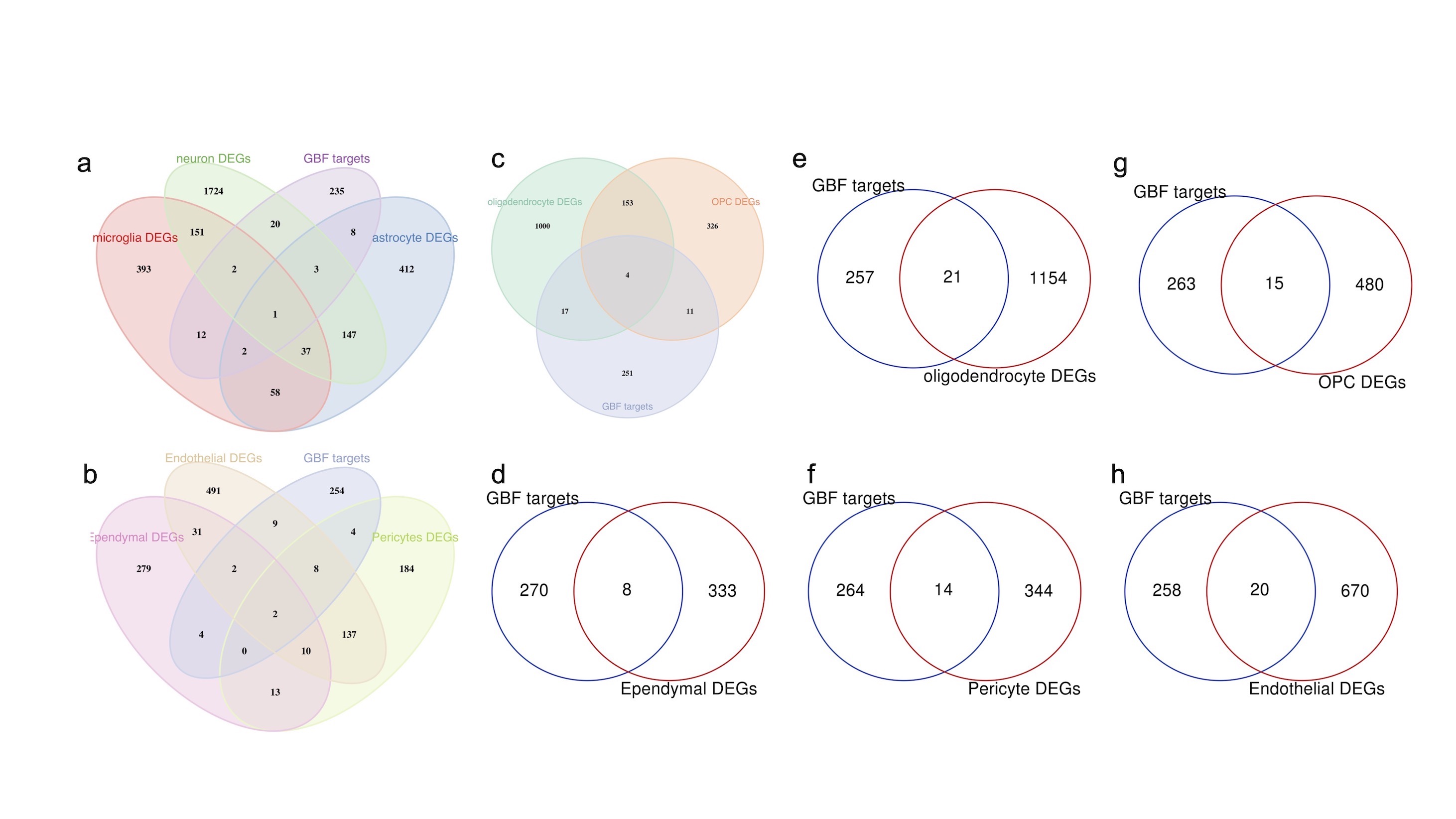

Supplement: Supplementary file 5 [file Image1.JPEG]

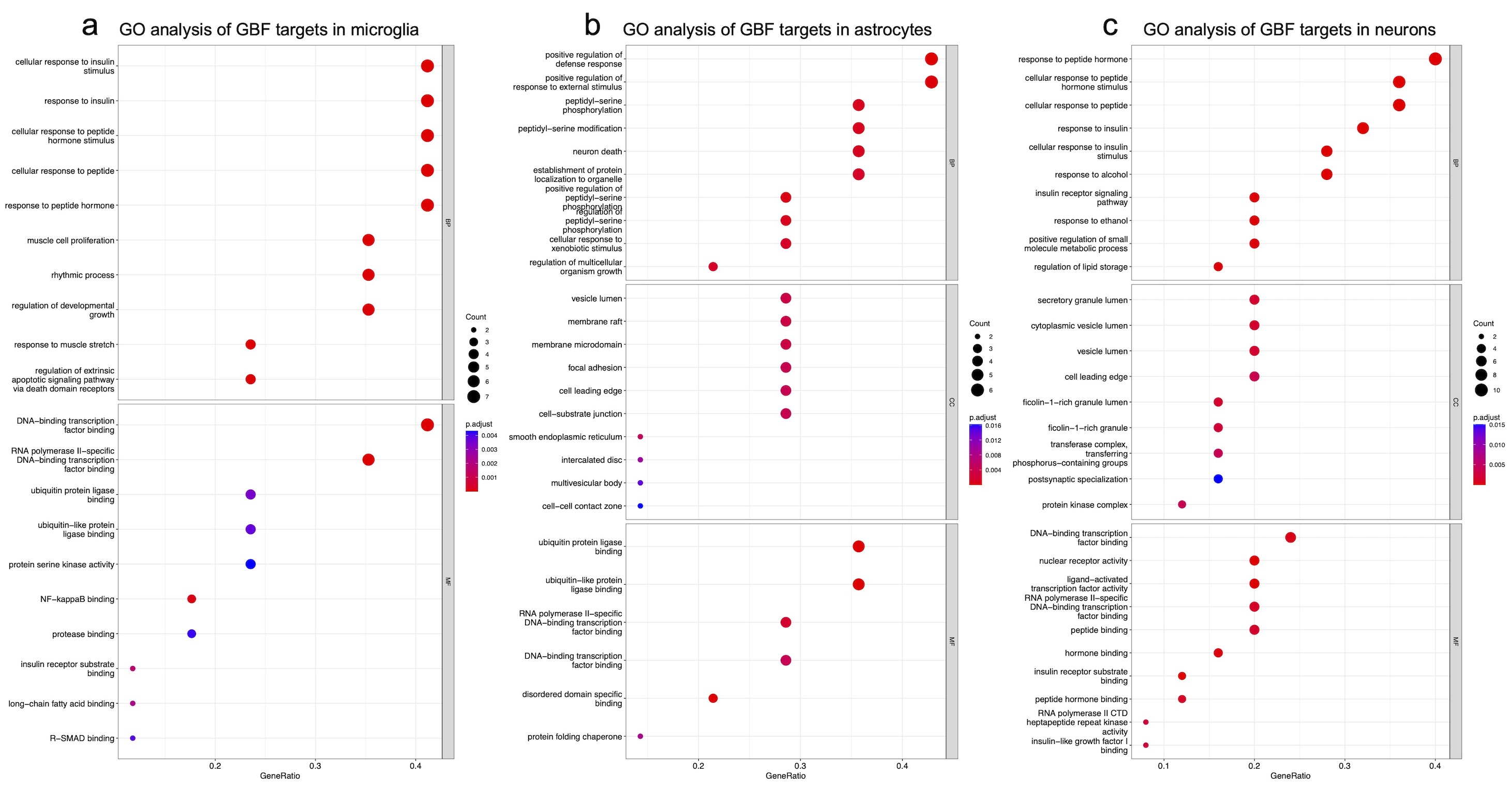

Supplement: Supplementary file 7 [file Image2.JPEG]
